# Effect of saccharomycin, a natural *Saccharomyces cerevisiae* biocide, on *Hanseniaspora guilliermondii* cells surface

**DOI:** 10.1080/07853890.2021.1896881

**Published:** 2021-09-28

**Authors:** Joana Calvário, Nelly Silva, M. Gabriela Almeida, Helena Albergaria, Peter Eaton, Anjos Macedo, Jorge Caldeira

**Affiliations:** a UCIBIO REQUIMTE FCT UNL, Caparica, Portugal; b Centro de Investigação Interdisciplinar Egas Moniz (CiiEM), Egas Moniz Cooperativa de Ensino Superior, Caparica, Portugal; c Unit of Bioenergy Laboratório Nacional de Energia e Geologia, Lisboa, Portugal; d LAQV REQUIMTE FC UPorto, Porto, Portugal; e LAQV REQUIMTE FCT UNL, Caparica, Portugal

## Abstract

**Introduction:**

During spontaneous wine fermentations, most of the non-*Saccharomyces* yeasts present in grape musts show an early decline in their population. It was traditionally assumed that *Saccharomyces cerevisiae* (*S.c.*) prevalence was due to the higher resistance of this species to ethanol. However, wine fermentations performed with single cultures of non-*Saccharomyces* strains showed that those strains could withstand much higher ethanol levels [1]. It was then found that *S.c.* (strain CCMI 885) produced antimicrobial peptides (AMPs) that are responsible for the early death of the non-*Saccharomyces* yeasts [2]. In previous work, we isolated, purified and sequenced those ntimicrobial peptides (AMPs) and found that they derive from the glyceraldehyde 3-phosphate dehydrogenase enzyme [3]. These GAPDH-derived AMPs compose the natural biocide secreted by *S.c.*, which we named saccharomycin, and are effective against sensitive yeasts both in its natural/isolated and synthetic form [4,5].

**Materials and methods:**

*Hanseniaspora guilliermondii* (*H.g*.) were grown from 8 to 48 h in YEPD, centrifuged and washed (ethanol 3% or 6%) prior to incubation with natural the *S.c.* AMPs, at 25 °C for 1 or 2 h. Surface of *H.g.* cells were observed by Atomic Force Microscopy (AFM).

**Results:**

AFM images of *H.g.* cells before and after (Figure 1) exposure to the AMPs show a significant changes on their surface.

**Conclusions and discussion:**

Analysis of the cell's roughness, reveals that untreated cells are smooth, unlike the treated with AMPs. Cell´s surface roughness increased upon AMPs contact by about 50% from 40.31(±12.87) nm to 58.01(±13.97) nm. Surface morphological details also indicates a destructive effect of saccharomycin on the *H.g*. cell wall.

**Figure 1.**
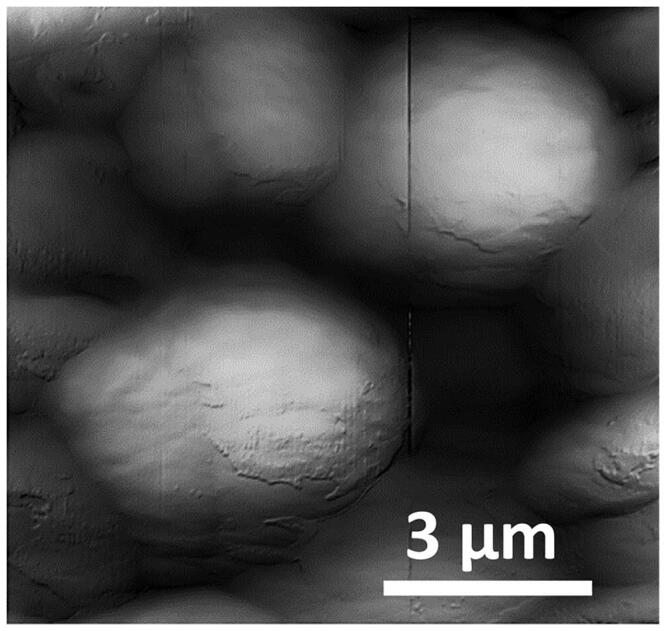
AFM image of H.g. cells after contact with the anti-microbial peptide (Saccharomycin) for 24 h, with roughness increase and surface rupture.
